# MEASURING BRAIN WAVES IN THE CLASSROOM

**DOI:** 10.3389/frym.2020.00096

**Published:** 2020-08-11

**Authors:** Nienke van Atteveldt, Tieme W. P. Janssen, Ido Davidesco

**Affiliations:** 1 Faculty of Behavioural and Movement Sciences, Section of Clinical Developmental Psychology and Institute Learn!, Vrije Universiteit Amsterdam, Amsterdam, Netherlands; 2 Department of Educational Psychology, University of Connecticut, Storrs, CT, United States

## Abstract

Brain researchers used to study the workings of the brain only in special laboratories at universities or hospitals. Recently, researchers started using portable devices that people can wear on their heads outside of the laboratory. For example, these devices allow researchers to measure the brain activity of students in classrooms, as they go through the school day. This sounds futuristic, and maybe also a bit alarming. In this article, we will explain what such devices do and do not measure—for example, they cannot read your mind! We will also explain how this kind of research can be useful to you and your classmates.

Have you ever heard about **brain waves** and maybe wondered what they are? In this article, we will explain what brain waves are, how they can be measured in the lab and in the classroom, and why it is interesting to measure them.

**BRAIN WAVES**

Cycles of electrical currents generated by groups of neurons that are active at the same time.

## EEG: MEASURING ELECTRICAL ACTIVITY IN THE BRAIN

The cells in your brain are called **neurons**, and your brain has roughly 86 billion of them. These neurons are very chatty, just like students in a classroom. Instead of using words, neurons communicate via tiny electrical signals that they generate. These signals go up and down in intensity, resembling waves: these are your brain waves. We can measure brain waves using a technique known as electroencephalography (**EEG**), in which small detectors, called **electrodes**, are placed on a person’s head [1]. Usually, all these electrodes (up to 256!) are held in place by a cap, although portable devices have recently been developed that use fewer electrodes, in fancier-looking headsets. EEG cannot measure the electrical activity of individual brain cells, because the electrical currents that any one neuron generates are too small. These currents can only be measured when many neurons transmit similar electrical signals at the same time. Imagine a music festival with thousands of people. When only one person claps, the band on the stage will not hear it, but when the whole audience claps at the same time, they surely will.

**NEURONS**

The cells in your brain that communicate with each other by transmitting electrical signals.

**EEG**

Electroencephalography, a technique in which small detectors, called electrodes, are placed on a person’s scalp using a cap or a headset. EEG measures the electrical activity of groups of neurons that transmit similar electrical signals at the same time.

**ELECTRODE**

A detector placed at the scalp, used in EEG to record the electrical currents generated by neurons in the brain.

## BRAIN WAVES: SLOW AND FAST

Brain waves vary in speed. You can think about slow brain waves as large waves in the ocean, moving a ship up and down, and fast brain waves as small ripples on the water’s surface. When we use EEG, we get a mixture of fast and slow brain waves happening at the same time.

**Figure 1:**
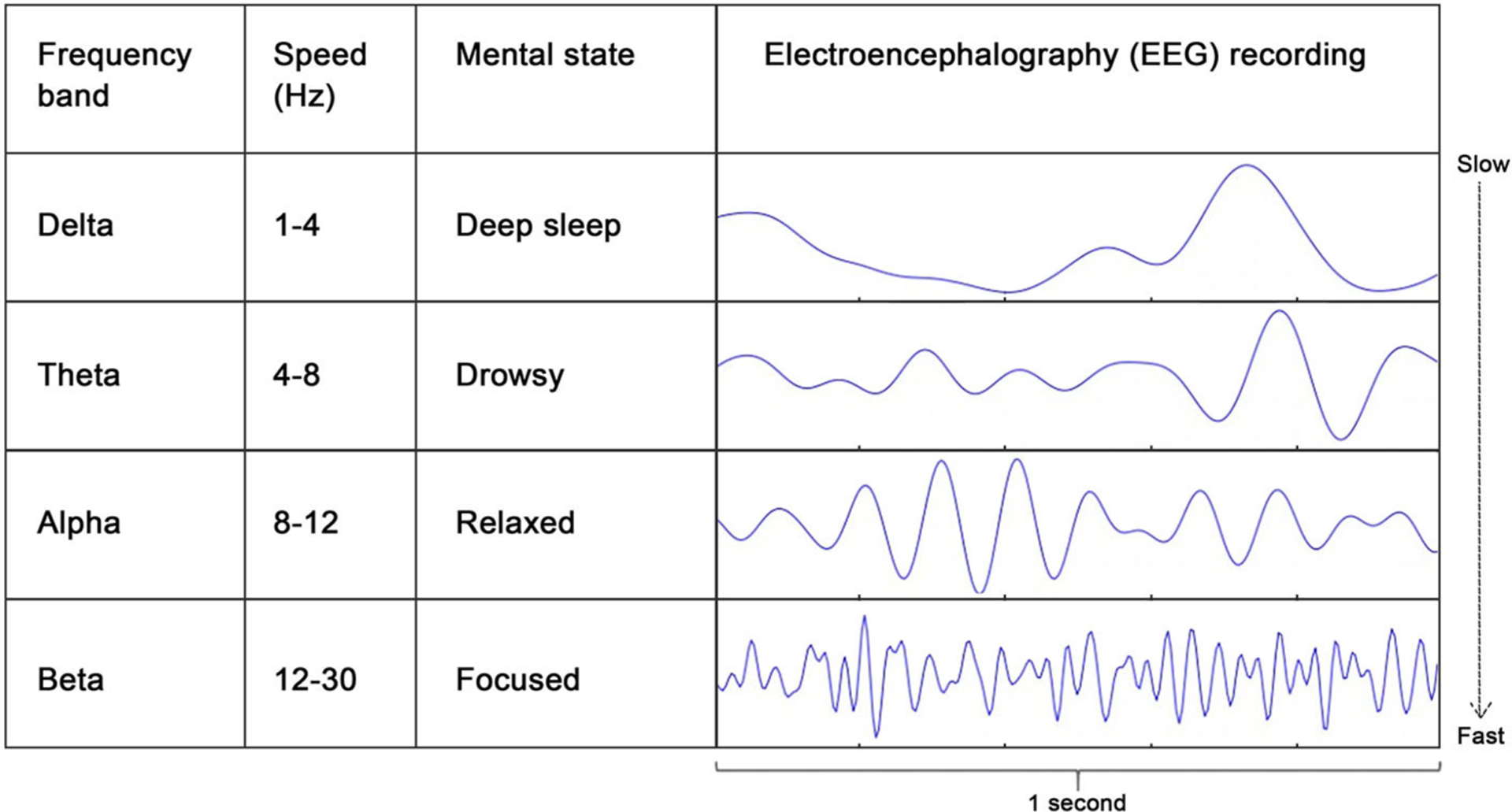
EEG frequency bands from slow to fast and how they relate to mental state. Brain wave frequency is measured in Hertz (Hz), which is the number of waves per second.

So why is this interesting? Imagine yourself early in the morning, not quite awake and still dreamy. If we measured your brain waves with EEG at that very moment, we would see relatively slow brain waves. Now imagine you are at school taking an exam, focusing intensely. In this situation, we might detect faster brain waves. These examples show that the speed of the brain waves is related to the state you are in. The speed of brain waves is called the **frequency**. We can identify different frequency ranges using EEG. For example, the Delta range corresponds to relatively slow brain waves that go up and down 1–4 times in a second, or 1–4 Hertz (Hz), which is the unit of frequency. Figure 1 shows an overview of frequency ranges (also called **frequency bands**) and how they relate to your mental state.

**FREQUENCY**

Speed of a brain wave; number of times a brain wave goes up and down in 1 s. The unit of frequency in Hertz (Hz); 1 Hz means one cycle per second.

**FREQUENCY BAND**

A range of brain wave frequencies that is associated with a certain mental state. For example, frequencies in the range of 1–4 Hz are called the delta-band, which is associated with deep sleep.

## BEYOND SLOW AND FAST: EVENT-RELATED POTENTIALS

Although EEG frequency bands are very interesting, not all questions can be answered by examining them. For example, what if you want to know how the brain understands the words you hear or how it controls impulses, like not hitting your younger sister if she drives you mad? For such questions, researchers analyze brain waves in another way: by calculating the event-related potential, or **ERP**. ERPs are the electrical brain responses to specific events, such as reading a word or controlling an impulse. In the ERP method, the parts of the EEG signal caused by these specific events are examined. To use this method, the EEG is recorded while the participant performs a computerized task that is specifically designed to study a certain function of the brain, for example impulse control.

**ERP**

Event-related potential, measured using EEG. ERPs are the electrical brain responses to specific events, such as hearing a sound or reading a word. In the ERP method, the participants perform a computerized task in which the specific event of interest is often repeated. The parts of the EEG signal caused by these events are averaged together. This averaging causes random brain activity to be averaged out and the relevant part of the EEG to remain; this is the ERP.

**Figure 2:**
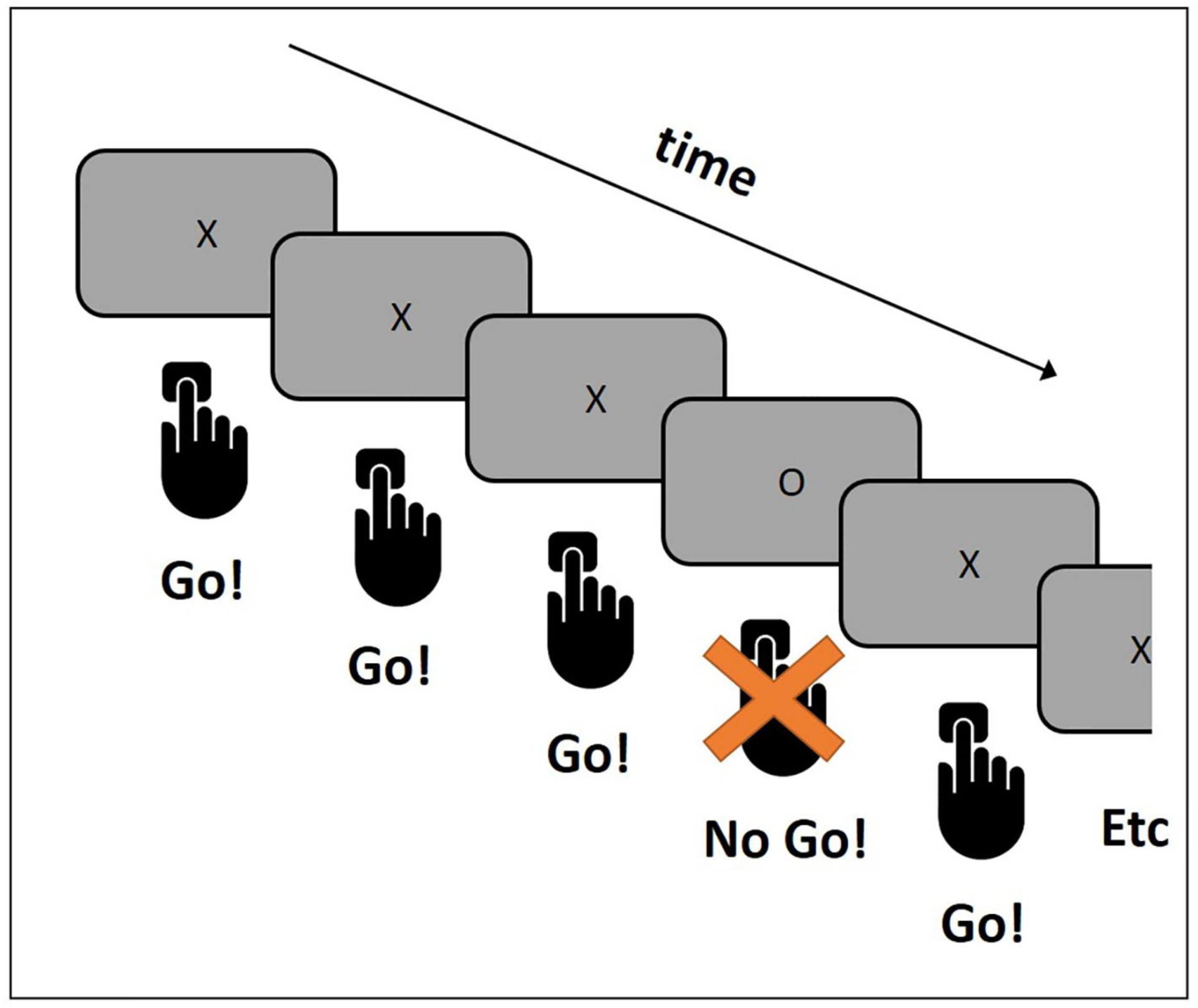
The Go/No-Go task. The letters X and O appear on the screen one at a time. The participants are asked to press the button ASAP when they see an X, and to NOT press the button when they see an O. The X appears very often and the O only occasionally. This makes it hard to inhibit the impulse to press the button when an O appears on the screen.

Here is a description of such a task, called a “Go/No-Go” task (Figure 2). Different letters appear on the screen, one by one. An “X” means “press the button” (Go!), and an “O” means “do NOT press the button” (No Go!). The “X” in this task is presented much more frequently than the “O,” so participants automatically prepare to respond whenever a letter appears on the screen—even an “O.” Participants need to control their impulse to press the button in the case of an “O.” When the task is over, the researchers examine the EEG recorded during the presentations of the X’s and O’s on the screen. Can you guess which letter they are most interested in?

Researchers are most interested in the EEG response to the “O”s, because this is when the participant needs to control the impulse to press the button. To examine the brain response to the “O”s, the researcher isolates the EEG response to each presentation of an “O” and averages all these responses together. The averaged EEG response to this specific event is the ERP, and it reflects the brain’s attempt to control an impulse. You can think about the process of calculating the ERP as a sieve, filtering out pieces of the EEG signal that are of no interest, leaving only the signals that researchers are the most interested in.

## THE LIMITATIONS OF LAB EXPERIMENTS

Scientists have learned a great deal about how the brain works from doing EEG and ERP experiments in laboratories. When we do such experiments, we usually measure brain activity when people perform computerized tasks. Such tasks are designed to measure a certain brain function, for example reading words, doing arithmetic, or controlling impulses. Usually, such laboratory tasks are quite different from things that we do in our day-to-day lives.

For example, think about the task with the frequent “X”s and rare “O”s used to study impulse control. Is this the same as controlling your impulses to move around or to chat with another student while your teacher is giving instructions? In the EEG lab, you would be sitting alone, in a quiet room, doing a task like pressing buttons and occasionally trying not to press a button. This lab experiment can tell us some things about how the brain controls impulses, but what does it say about how children deal with their impulses at school? This is a limitation of lab experiments: they measure brain activity in rather unnatural situations [2].

## USING PORTABLE EEG IN THE CLASSROOM

Another aspect of human behavior that is difficult to study in a laboratory is how people interact with one another, for example, the way students interact with each other in school. Laboratory experiments are extremely limited in answering this question, but recent developments in portable EEG now allow scientists to conduct brain research outside of the laboratory.

**Figure 3:**
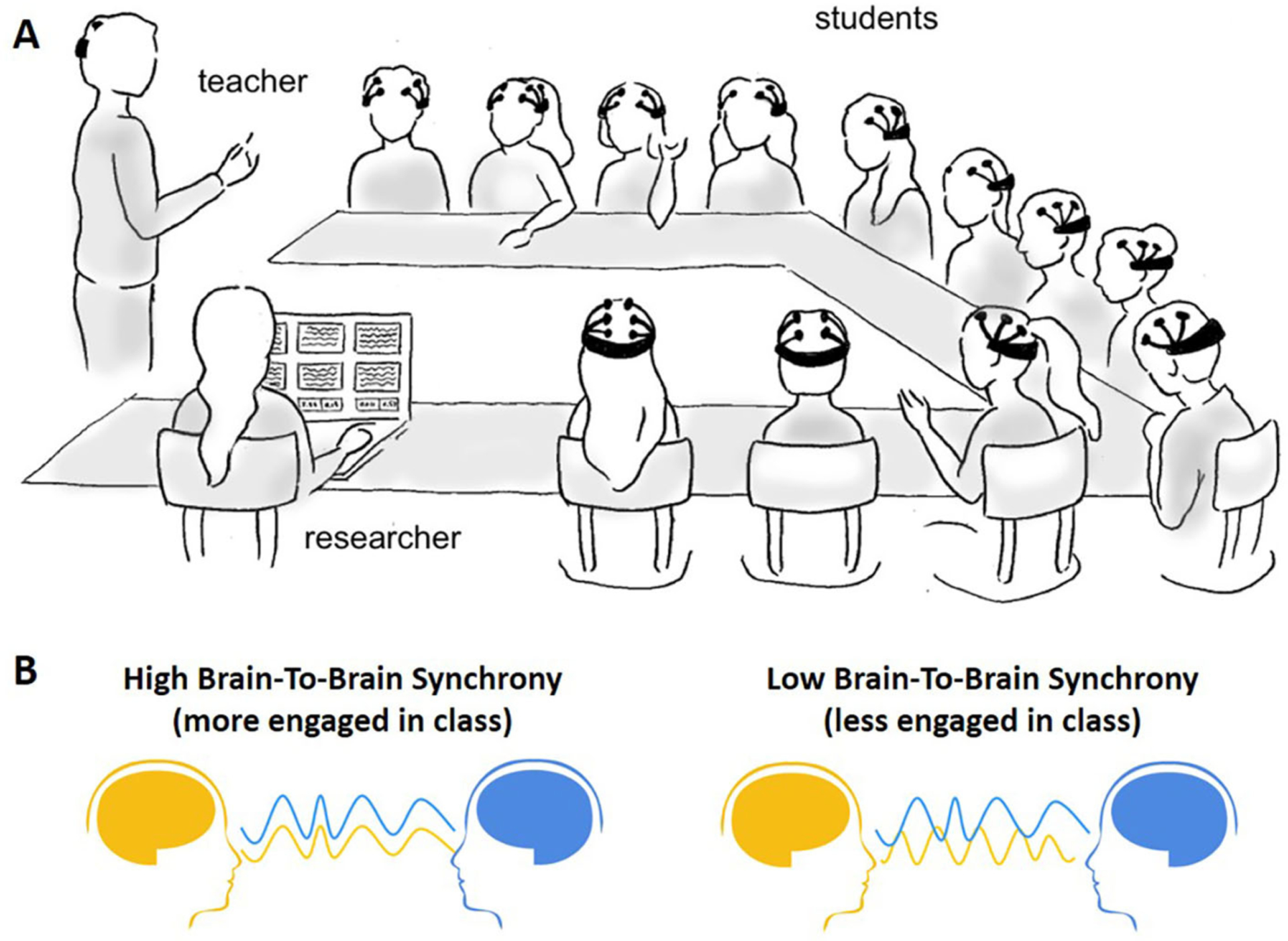
**(A)** EEG can be used to measure the brain waves of students in a high school classroom (from: Dikker et al. [3]). **(B)** Students’ brain waves can show high synchrony with other students, which was found for students that were more engaged in class (left). Low synchrony with other students (right) was found for students who were less engaged.

This is exactly what a team of researchers at New York University did recently [3]. They partnered with a local high school and measured the brain activity of a teacher and a group of students during 11 biology lessons (Figure 3A). In each lesson, the students participated in different learning activities, such as lectures, instructional videos, and group discussions. The researchers found that, during these classroom activities, students’ brain waves were in **synchrony**. In other words, their brain waves went up and down together, in sync. Even more interestingly, students who reported being more engaged in class were even more in sync with the other students (Figure 3B).

Portable EEG devices are exciting because they can be used not just for research, but also for teaching purposes. In “BrainWaves,” a neuroscience high school program that was developed at New York University, students use EEG to learn about their own brains, and about how neuroscience works. Students work with a scientist to develop their own research projects. For example, they can use EEG to explore how the brain responds to images of famous and non-famous faces, or how listening to music affects our ability to concentrate.

**SYNCHRONY**

When brain waves go up and down together. This can either be within one brain (e.g., brain waves from different parts of the brain) or between brains. This latter example is called brain-to-brain synchrony.

Portable EEG was not invented to replace laboratory EEG research. Rather, it complements lab research by providing insights on brain processes in day-to-day situations. But the advantage of studying the brain in a more natural setting comes with some trade-offs. The quality of the data collected by portable EEG is not as high as the data collected in the lab, because portable devices have far fewer electrodes and participants move around more. Also, the environment outside the lab is not under the researcher’s control, so the experimental results might be more difficult to interpret.

## DOES THIS SOUND LIKE SCIENCE FICTION?

So, after reading all of this, what do you think? Would you be interested in wearing an EEG device in your classroom, or do you find this thought a bit scary? Well, to reassure you, so far portable EEG only provides a general measure of brain activity. EEG certainly cannot read your mind. So, you do not need to worry that researchers or your teacher could read your thoughts if you ever put on one of these EEG devices at your school. We want to reassure you that mind reading is still science fiction!

Some commercial companies that make and sell EEG devices do claim that EEG can be used to monitor students, by reading the strength of different brain waves and decoding this into “concentrated” or “distracted.” We do not think this is a very good idea, for various reasons. First, we need to do much more research before we understand enough about what the EEG signals mean in terms of brain functions. Second, students do not necessarily need to be concentrating all the time. We know that the brain also needs some time to rest, and mind wandering can actually be useful for learning [4].

## CONCLUSION

Portable EEG devices offer some great opportunities, such as the ability to study how the brain works in natural environments, like classrooms. Study of the brain in natural situations can especially benefit our understanding of social interactions, as portable EEG can be used to measure the brain activity of several people at once, while they are interacting with each other. Moreover, portable EEG can also help students to better understand how the brain works. However, science advances in small steps, so let us leave mind reading for science fiction films, and in the meantime discuss whether we will ever want that to be a reality [5].

## YOUNG REVIEWERS

THE SCHOOL FOR SCIENCE AND MATH AT VANDERBILT, AGES: 14–15

We are a class of students from all over Nashville, who come together once per week at Vanderbilt to learn more about science, technology, engineering and mathematics. We conduct experiments in our classroom and in labs on campus!
